# The Dynamics of Doctor-Patient Communication During Remote Consultations: Qualitative Study Among Norwegian Contract General Practitioners

**DOI:** 10.2196/57679

**Published:** 2025-03-27

**Authors:** Børge Lønnebakke Norberg, Bjarne Austad, Eli Kristiansen, Paolo Zanaboni, Linn Okkenhaug Getz

**Affiliations:** 1 General Practice Research Unit Department of Public Health and Nursing Norwegian University of Science and Technology Trondheim Norway; 2 Norwegian Centre for E-health Research University Hospital of North Norway Tromsø Norway

**Keywords:** remote consultations, digital consultations, telemedicine, eHealth, communication, safety, general practice, family medicine, focus groups, telehealth, digital health, relationship, patient-physician, general practitioner, thematic analysis, qualitative analysis

## Abstract

**Background:**

Patient consultations in general practice are undergoing a digital transformation, embracing diverse modalities such as video, text-based, and telephone consultations. The quality of communication in medical consultations is pivotal for successful outcomes, necessitating a comprehensive assessment of the impact of this transformation on doctor-patient communication and interaction.

**Objective:**

This study aims to explore general practitioners’ (GPs’) perspectives on how the communication between Norwegian contract GPs and patients has been affected by the large-scale implementation of remote consultations following the onset of the COVID-19 pandemic.

**Methods:**

Five focus groups, comprising 18 purposefully recruited GPs from diverse settings and geographical regions in Norway, were carried out in 2022. We applied thematic analysis guided by the framework proposed by Braun and Clarke.

**Results:**

Six themes resulted from the analysis. First, suitability regarding remote communication is context-dependent: knowing the characteristics of the patient as a person and the clinical relationship is more important than the reason for contact or type of health problem—even more so than during ordinary physical consultations. Second, remote consultations favor a demarcated communication style, “keeping things simple—the one-problem approach,” which can increase work effectiveness. Third, a downside of such effective minimalism is that the uncritical use of remote consultations may undermine the quality of care. Communication becomes too transactional, limiting the chances of addressing more implicit and complex issues, with the risk of missing vital information. Fourth, remote modalities can help engage hesitant and vulnerable patients. Fifth, GPs make communicative trade-offs in the name of continuity to be able to maintain relationships with patients they see as vulnerable or fugitive. Finally, there are advantages and dilemmas stemming from text-based consultations. Although they offer benefits such as multimedia-enabled patient expression and sharing of digital information, some concerns include the risk of information loss through triage errors, managing informal language, and ending chat-like interactions between patients and doctors.

**Conclusions:**

The implementation of remote consultations has many effects on clinical interaction and communication. Although these modalities can enhance efficiency, there is a discernible risk of compromised retrieval of essential information and unvoiced problems, potentially resulting in unintended consequences. The preservation of continuity of care emerges as a pivotal strategy to mitigate some of these challenges.

## Introduction

### Background

Strong clinical relationships and effective communication during general practitioner (GP) encounters are considered crucial to the overall quality of primary care [1-5]. This importance extends to remote consultations [6-11], which have seen rapid and widespread adoption in response to the challenges posed by the COVID-19 pandemic [12-15]. This global shift has altered the dynamics of medical consultations [16,17] and had a profound impact on the substance and efficacy of communication between GPs and their patients [8,11,18-24].

Remote communication introduces both novel opportunities and challenges in clinical practice [6-11,25-27]. Remote consultations inherently elevate accessibility, offering efficient, environmentally friendly, and positively received solutions for patients [12]. However, the “gold standard” patient-centered communicative approach was developed before the era of remote communication. In other words, the extensive implementation of digital modalities disrupts established practices in ways that have been hard to anticipate [28-33]. Recent research indicates that remote consultations may compromise communicative and diagnostic quality, posing suboptimal clinical evaluation and the risk of overlooking critical information [24-29]. Furthermore, remote communication might also curtail the handling of social determinants of health [8,12,31].

Seen in isolation, each type of remote communication is associated with documented strengths and weaknesses [24-28]. Telephone consultations are deemed appropriate for noncomplex, relatively simple contacts, including triage [34-37], but are susceptible to limitations, including simplistic dialogue, challenges in conveying emotions and sensitive messages, and unsatisfactory clinical assessment [29,32,38-41]. Video consultations, while comparable with telephone consultations in terms of duration, content, and quality [38-41], still present challenges in establishing a doctor-patient relationship, with patients reporting feelings of rush and difficulties in turn-taking in the conversation [8]. Recent publications nevertheless reveal strengthened alliances between patients and physicians in video consultations, compared with the pandemic context [20,21,38,39]. Video consultations are found to be superior to telephone consultations for more demanding triage involving visual evaluation, certain psychological issues, discussing test results, and addressing changes in medication [20-22,38-41]. Text-based consultations, often recognized for their brevity, can reproduce crucial components of face-to-face consultations, encompassing history-taking, investigation, and final assessment [42-46]. Despite their streamlined nature, text-based consultations may generate additional workload for GPs, stemming from unfiltered access and heightened demands [28,46,47]. Some practitioners have noted a decline in the doctor-patient relationship linked to text-based consultations [44-46]. However, studies have also shown instances of strengthened therapeutic relationships [24,45] and improved accessibility over time, especially for individuals dealing with long-term health conditions, attributable to enhanced availability [30,31,42-46].

Much of the existing research on remote consultations stems from contexts where services were only piloted or during the COVID-19 pandemic when use was forced upon both clinicians and patients [12-15]. Contextual factors evidently play a decisive role [48], and consensus on communicative changes or challenges associated with remote consultations remains elusive [8-11,25,45,49,50].

Consequently, it seems essential to continuously observe the impacts of the digital transition on communication dynamics in remote consultations across different contexts, including contractual GP schemes. How will the digital transformation settle into the “new normal” after the resolution of the pandemic [7-9,18,27]? This study examined remote consultations as an integral part of the daily routines of contract GPs in Norway in a more or less normalized postpandemic situation without societal restrictions. The study’s aim was to explore the impact of remote consultations on interaction and communication between GPs and patients in this context.

### Study Context

#### Remote Consultations

This study focuses on 3 types of remote consultations: video, telephone, and text-based consultations. Video and telephone consultations are synchronous interactions occurring between doctors and patients on various devices, including phones, tablets, or personal computers. Text-based consultations occur as asynchronously written dialogues between the patient and the doctor, which are initiated or prearranged with the patient. Norwegian GPs are expected to respond to patient-initiated text-based consultations within 5 working days. Text-based consultations normally take the form of a freely formulated essay-type request potentially supplemented by a predefined questionnaire filled out by the patient.

Remote consultations in Norway peaked at nearly two-thirds of all consultations during the COVID-19 lockdown and have since stabilized at 20%-25% of all GP consultations in 2024 [51]. The prevalence of video consultations remains relatively low [52], while telephone and text consultations have sustained high utilization postpandemic, influenced partly by the reimbursement system [53].

In Norway, 80% of all patients use the national health portal Helsenorge [54] for electronic booking of appointments, electronic prescription renewals, electronic contact with the GP office for nonmedical inquiries, and text consultations for clinical inquiries with the GP. The use of remote consultations is not mandatory for GPs, so each GP can decide whether to offer text, video, or telephone. Patients can typically book physical, video, and telephone consultations on the web. Technical equipment for video consultations is available through both private and public external providers and is becoming more integrated with patient record systems.

#### The Norwegian Contract GP Scheme

Norway’s health care system is grounded in the principles of universal access, decentralization, and continuity of care [55]. Since 2001, all Norwegian citizens have the option to enroll (or change enrollment) with a GP, a choice exercised by 99% of the population. This scheme is designed to ensure continuity of care, with GPs serving as coordinators of municipal services and gatekeepers to specialized services. Norway’s 5391 (per May 2024) contract GPs are each responsible for a defined patient list (typically around 1000 citizens or patients) from 8 AM to 4 PM on weekdays and often participate in evening and night emergency shifts in the municipality or region.

Being a specialist GP is a unique specialty in Norway, similar to, for example, ophthalmology or psychiatry. It entails a 5-year program of specified clinical services in accredited practices or institutions, combined with defined learning objectives such as practical procedures, courses, and guidance. The specialty certification must be reaccredited every 5 years [56].

Contract GPs’ income systems vary to some extent. The majority receive a fixed annual contribution per patient to help cover practice expenses. Each consultation is reimbursed by a set fee, with additional reimbursement for specified procedures, for example, electrocardiogram testing, surgical procedures, and conversational therapy. Furthermore, GPs receive out-of-pocket fees from patients, typically around 230 NOK (US $21) per consultation (whether physical, text, telephone, or video), up to an annual maximum per citizen for essential medical expenses (currently 3165 NOK = US $287). Expenses above this are fully covered. The provision of remote consultation services is not mandatory for contract GPs, and a small fraction disables 1 or more of these options.

Alongside the well-developed publicly regulated system (contract GP scheme), a few fully private actors offer regular medical care at the patient’s expense, and some private digital care companies offer video or text consultations. Private medical insurance is limited in Norway but is increasing.

## Methods

### Study Design

We conducted a qualitative focus group study among Norwegian contract GPs. Focus groups are deemed suitable for exploring attitudes, experiences, and areas with limited prior knowledge, and they allow for the exploration of tentative and conflicting views [57-59]. The Standards for Reporting Qualitative Research Reporting Guidelines [60] have been consulted in the reporting process (checklist in Multimedia Appendix 1).

Our research team comprised 2 experienced contract GPs (BLN and BA) with substantial experience with remote consultations, a former GP who is now a full-time professor in behavioral sciences in medicine (LOG), and 2 experienced digital health researchers (PZ and EK) with backgrounds in engineering and economics, respectively. LOG, PZ, and BA hold PhDs, with LOG and PZ serving as professors. LOG and BA are seniors in a general practice research unit, introducing solid academic perspectives on general practice, including the documented relevance of continuity of care, patient-centered communication, and relational trust. BLN has extensive experience as a lecturer in clinical communication for medical students. All senior team members had previous experience with qualitative research.

### Recruitment Procedure

We used purposive sampling to include experienced contract GPs with familiarity with remote consultations to participate in the focus groups. We approached participants or educational groups by phone and mail from diverse geographical regions, including both urban and remote municipalities, as well as participants with varying income systems in the contract GP scheme (fixed salary, subsidized positions, or paid per capita).

Participants were recruited in 2 ways: 3 groups comprised GPs from preexisting postgraduate educational groups associated with the formal recertification system for GPs in Norway. Such groups are compulsory and meet regularly to engage in structured activities and discussions of general practice topics. These GPs were professionally acquainted beforehand, something we considered to be a facilitating factor. The remaining 2 groups included experienced, opinionated, and nuanced GPs, individually recruited through the research team’s professional network.

All but 1 invited GP agreed to participate. Some of the participants were known to members of the research team beforehand, but there were no personal bonds likely to impact the integrity of the data. The initial plan was to conduct 4 focus groups, but a fifth group was added after preliminary analysis of groups 1-4 to further enhance information power [57].

Following the initial invitation, participants received written information outlining the project’s purpose, data handling, and withdrawal procedures (Multimedia Appendix 2). No exclusions or dropouts occurred.

### Data Collection

The focus group interviews were conducted a while after pandemic restrictions had been lifted in Norwegian health care, between January 15 and April 20, 2022. Three interviews were conducted physically in local medical center meeting rooms, while 2 were carried out remotely on the platform teams, involving GPs from rural regions and other parts of Norway. Interviews were conducted in Norwegian.

Prior to the recorded interview sessions, participating GPs were reminded that participation was voluntary before they signed informed consent (Multimedia Appendix 2). Participants were instructed to carefully anonymize all patient-related experiences. The participants provided anonymized information about themselves and their practice.

During the interviews, moderators BLN and BA used a semistructured interview guide, collaboratively designed by BLN, BA, and LOG, featuring flexible, open-ended questions (guide in Multimedia Appendix 3). The team’s previous publications on remote consultations [4,20] have significantly contributed to a robust database of physician responses, which in turn informed the thematic focus of the interview guide. The guide was pilot-tested with 2 GPs and 2 nonmedical PhD students involved in qualitative research.

The focus group interviews lasted 95-120 minutes. They were audio-recorded and transcribed verbatim. The process remained open for corrections and additions. One follow-up interview provided in-depth insights into the lack of emotional exchange during remote consultations. Participants were compensated approximately 1000 NOK (US $91), which is about half of the compensation for 2 hours of medical consultant work.

### Data Analysis

Reflexive thematic analysis, following the approach proposed by Braun and Clarke [57-59], was used to establish patterns of meaning across the data. Initially, LOG and BLN conducted individual analyses: LOG manually and BLN using NVivo (QSR International [Lumivero]) for digital processing. Along the way, BLN and LOG compared and discussed results, followed by joint meetings with BA. The main analytic strategy was inductively derived from the data, albeit influenced by the researchers’ professional situatedness. Some elements of the analysis were deductively guided by theory related to the phenomenon of trust. Subsequently, preliminary themes and candidates for final themes were shared with the entire research team, supported by 2 external project collaborators (see the Acknowledgments section). At this stage, theme development underwent a major revision. This paper focuses on microperspectives (here defined as doctor-patient communication), leaving meso- and macroperspectives (ie, addressing the impact of remote consultations on GP offices and the health care system at large) to a forthcoming publication. In the final stages of analysis and writing, exchanges among all authors aided in challenging individual biases and presuppositions, enhancing the validity and relevance of our findings.

### Ethical Considerations

All participants signed an informed consent to participate. This study was conducted among health care workers, involving no patients or sensitive health care information. According to the Norwegian Act on Medical and Health Research §2 and §4, the study did not require approval from the regional ethics committee. Interview data were safely secured in accordance with national and institutional regulations. In the transcription and publication process, personal and demographic information was anonymized to prevent indirect identification of the GPs. The procedure for handling the data was approved by the Norwegian Center for Research Data NSD/SIKT (reference: 531672).

## Results

### Participant Characteristics

A total of 18 GPs participated in 5 different focus groups, each consisting of 3-5 members. They all had substantial experience using all forms of remote consultations, but 3 had limited their access to text consultations due to high demand. An overview of participant characteristics can be found in Table 1.

**Figure 1:**
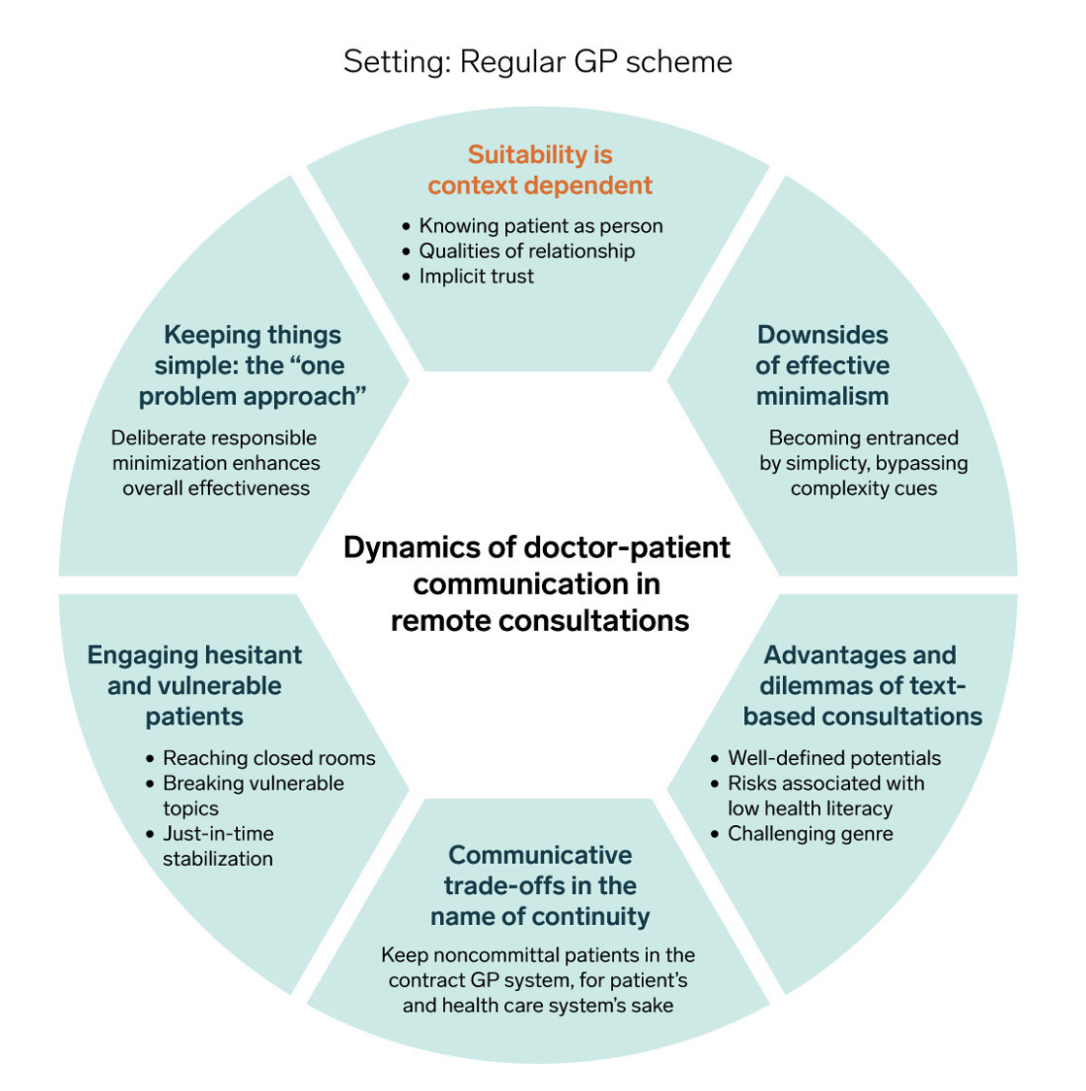
Overview of the themes developed from 5 focus group discussions. GP: general practitioner.

Analysis of the focus group discussions on the communicative aspects of remote consultations unveiled a nuanced landscape marked by both advantages and disadvantages, giving rise to 6 themes, summarized in Figure 1.

**Table 1:** Characteristics of participating GPs.

Number^a^	Age category (years)	Sex	Use of video, telephone, or text consultations	Specialist in general practice	Geographical location	List size (approximate)	Interview format
1	30-40	F^b^	Video, text, and telephone	Under specialization	Rural	500	Digital
2	30-40	M^c^	Video, text, and telephone	Yes	Rural	1000	Digital
3	40-50	F	Video, text, and telephone	Yes	Rural	500	Digital
4	50-60	F	Video, text, and telephone	Yes	Rural	600	Digital
5	50-60	F	Video, text, and telephone	Yes	Urban	1100	Digital
6	50-60	F	Video, text, and telephone	Yes	Urban	1300	Digital
7	40-50	F	Video, text, and telephone	Yes	Urban	1200	Digital
8	50-60	F	Video and telephone; text turned off because of high demand	Yes	Urban	1200	Digital
9	40-50	M	Video, text, and telephone	Yes	Urban	1000	Physical
10	60-70	M	Video, text, and telephone	Yes	Urban	900	Physical
11	40-50	F	Video, text, and telephone	Yes	Urban	800	Physical
12	40-50	F	Video, text, and telephone; limits access to text because of high demand	Yes	Rural-urban fringe	900	Physical
13	40-50	M	Video, text, and telephone	Yes	Rural-urban fringe	1200	Physical
14	40-50	F	Video, text, and telephone	Yes	Rural-urban fringe	900	Physical
15	40-50	M	Video, text, and telephone	Yes	Urban	1500	Physical
16	50-60	M	Video, text, and telephone	Yes	Some urbanity	1300	Physical
17	60-70	M	Video, text, and telephone; limits access to text because of high demand	Yes	Rural-urban fringe	1200	Physical
18	30-40	M	Video, text, and telephone; limits access to text because of high demand	Yes	Rural-urban fringe	800	Physical

^a^Numbers 1-4, 5-8, 9-12, 13-15, and 16-18 represent the 5 different focus groups.

^b^F: female.

^c^M: male.

### Suitability is Context-Dependent

A dominant view in all focus groups was that the applicability of remote consultations to communication between GPs and patients is largely independent of the reason for contact or specific diagnoses. Suitability largely depends on the doctor-patient relationship, the doctor’s knowledge of the patient’s medical history, and the patient’s ability to formulate his or her concerns concisely. In many ways, this theme has an overarching character, connecting and informing the subsequent themes that manifested from our analysis, as shown in Figure 1.

When you know the patient well, it is easier to agree on a plan...Background knowledge means a lot for an assessment.

ID6

One GP quoted the influential physician Sir William Osler (1848-1919) [61]: “It is much more important to know what sort of a patient has a disease than what sort of a disease a patient has.”

The sentiment among GPs resonated with the idea that:

Topics in consultations are often interconnected, mutually influencing each other [...]. A troubled marriage can manifest in several ways, and the need for sleeping pills may conceal significant underlying information.

ID16

The participants emphasized that both verbal communication and clinical examination and treatment via video were surprisingly feasible, especially when relational trust had been established between the GP and the patient. For example, a GP who had previously prescribed medication to a young patient with chronic urticaria recounted how it was easy to calm and instruct her over video as she needed to inject the drug:

She posed in shorts on her kitchen chair, and she was able to insert this medication subcutaneously without any problem.

ID5

### Keeping Things Simple—the “One-Problem Approach”

The GPs seemed to agree that remote communication has some fundamental qualities that differ from physical communication. Without any specific preparations or instructions from the GP’s side, the modality appeared to narrow things down to what the GPs called a “one-problem approach.” This phenomenon could often be perceived as beneficial:

The best thing about remote consultations is that they go quickly and smoothly, and you stick to the problem. Concise...it stops everything outside the original theme.

ID9

Furthermore, participants described that remote consultations, in general, appeared suitable for (presumably) demarcated problems with negligible risk of missing clinically relevant issues or risk factors. Therefore, they can be performed more quickly. In such instances, GPs experienced that remote consultations represented “good enough” medical work from a here-and-now perspective.

A benefit of keeping things simple relates to worried patients who have a habit of overseeking regular consultations. GPs have noted that even brief remote consultations can “work miracles” (quote from a participating GP) for these patients and reduce the number of time-consuming physical consultations.

Remote communication tends to have a transactional quality that seems to bypass implicit expectations of relational care. Once a distressed patient has waited for a physical consultation and perhaps traveled a long way, expectations can build up, and the GP can sense an obligation to spend more time with the patient as a sign of care and respect. This phenomenon rarely applied to remote consultations:

When you take that video, it is a bit more acceptable to...keep simple things simple.

ID2

However, the benefits of this approach were balanced by concerns about potential downsides, such as overlooking clinically significant aspects.

### Downsides of Effective Minimalism

Consultations characterized by the typical “one-problem approach” apparently entailed diminished cognitive and emotional demands on the GP in a here-and-now perspective, thereby contributing to welcomed work intensity variation during the day. However, the discussions of new potentials did not last long in any of the focus groups before a certain ambivalence and skepticism started to surface. GPs described a risk of being carried away by apparent simplicity, missing clinically significant aspects of their everyday work as they knew it from years of physical consultations. Uncritical use of remote consultations may undermine the quality of care:

It can easily take off in a spiral of efficiency—and then it quickly becomes a dangerous disadvantage.

ID11

The discussions revealed that the most evident pitfalls of remote consultations represent the flip side of the reported advantages. Participants described how the experienced efficiency could turn into a spiral of unintended minimalism:

If the GP makes too much use of remote aids and don't take consultations physically...one can imagine that the doctor fixes things (way) too quickly….

ID8

At a more specific level, several GPs were worried that in the flow of efficiency, relevant and essential information associated with the patient’s reason for contact remained out of sight and unaddressed:

You might miss these things on remote consultations. It can be central to realize that there is something else …something unsaid or unwritten.

ID17

GPs shared experiences pertaining to typical communicative qualities of remote consultations. Compared with physical encounters, communication becomes more linear, literal, and business-like. Information residing between the verbal lines seems to go unnoticed or remain unaddressed:

It is like an interpreter call…because it has become such an additional filter in between.

ID14

Keywords regarding what might easily be lost during remote consultations were context and cues, referring to relational and emotional factors that affect the symptom picture and have relevance for effective resolution:

Have you noticed? No one ever cries on video! ….

ID16

If contextual insight and emotional cues go unnoticed, the chance of an effective problem solution diminishes. This hampers the GPs’ ability to reveal serious illness. It is, therefore, not just about the number of problems that are lost, they elaborated, but more about the range of dimensions lost in communication:

I had a patient on video who had difficulty sleeping and wanted sleeping pills...I did not prescribe them. I rather wanted to find out what his life was really like and invited him to have a proper [physical] conversation […] Remote consultations may also generate 4-5 extra consultations for extending sick leave because the patient does not want to talk about his terrible boss on a remote consultation.

ID16

GPs recognized a distinct ritual associated with traditional, in-person consultations—a ritual that, for better or worse, imbues the medical encounter with a sense of uniqueness. Traditional health care requires patients to travel, wait, meet the doctor, and possibly undergo physical tests. Remote consultations eliminate these steps, saving time and seamlessly fitting into the patient’s daily routine. Participants elaborated that in remote GP appointments, patients often seem less mentally focused. Furthermore, such consultations are often initiated for minor complaints. These are seen as potential drawbacks. To minimize these drawbacks, some GPs aimed to meet patients physically at an early stage:

I like to start with a physical consultation to sense the non-verbal clues and include the big context.

ID15

In relation to video consultations, GPs described patients’ casual use of mobile cameras, simultaneously multitasking during all kinds of transportation and errands:

It is quite common, we have video consultations while the patient is driving, lies in bed or is all over the place.

ID14

GPs exhibited ambivalence toward patients’ multitasking while in remote consultations, experiencing a spectrum from frustration to recognizing occasional valuable moments. However, they explicitly appreciated their own multitasking opportunities. The pandemic prompted a shift in how GPs use eye contact during video consultations, transitioning from its prioritization to a more flexible approach amidst multitasking demands. This adaptation mirrored the younger generation’s informal use of social media, offering GPs a sense of freedom while acknowledging both the advantages and drawbacks.

The traditional face-to-face consultation was seen to strengthen the therapeutic alliance and give the GP a better understanding of the totality of the patient as a person. Overall, it appeared that most GPs remained alert to the potential side effects of “pleasing the patients” too much. Offering all patients who are reluctant to attend a physical consultation a choice of remote consultations as an easy way out might ultimately undermine these patients’ abilities to engage in trustful human relations and face the challenges of everyday life in a physical community. Still, none of the focus groups reached a clear consensus as to when or how the physical encounter is clearly to be preferred for therapeutic reasons.

### Engaging Hesitant and Vulnerable Patients

In all focus groups, GPs discussed how remote consultations may facilitate the establishment of new relationships. Examples include socially withdrawn patients and young people with high digital competence who have little experience with defining their own problems and seeking formal health care:

I have several patients with autism-like diagnoses or personality disorders with relational issues, who prefer to communicate remotely—by text, telephone, or video—rather than sitting in the waiting room for a physical appointment. They communicate better and seem to profit from these new services.

ID13

Another communicative gain was referred to by some participants as an “ice-breaking” effect, facilitating the sharing of sensitive information that, for some patients, could be harder to address in a face-to-face consultation:

...I do not necessarily think they would have been able to say these things when they were sitting here, right in front of me in a chair. So, sometimes that distance on video can make it easier to bring things up, eventually leading to a physical consultation.

ID3

Specific examples of sensitive subjects that might sometimes emerge due to, and not despite, the digital modality involve histories of sexual abuse:

It was difficult regarding trust initially…. But then a nice little dialogue started on text-consultation. A few small questions. So, in a way, I understand that she was trying to say something important [about abuse]. And then we go on…finally arriving at what was really the case. And this is a new unique way of working.

ID14

Some of the GPs had experienced that remote consultations were used by patients with suicidal thoughts that might not otherwise contact the health care system:

I talked to him in the car a good distance from his home and office...When he talked about his suicidal thoughts and how he might end up taking his own life, he liked to sit alone in his car. So, in a way, he set the conditions for being protected…

ID9

### Communicative Trade-Offs in the Name of Continuity

Although the GPs agreed that communication during remote consultations entailed a risk of losing vital symptoms and unvoiced problems, they shared experiences of how they would sometimes deliberately stretch their limits and agree to perform remote consultations simply to avoid losing young, immature, or otherwise vulnerable, noncommittal patients to competing commercial digital doctors. The GPs thought that such actors might not provide adequate, affordable, and high-quality services for complex patients in the long term because they would lack continuity of care. As one GP said about such patients, with reference to the contract GP scheme:

I work hard to “keep them in the system.”

ID7

An additional reason why the GPs tried to avoid the involvement of other digital doctors was that such consultations tended to create double work and even more additional work, as the contract GP might typically be contacted anyway and have to sort out the status of a process initiated by an unknown colleague:

I would rather help them myself than get help from someone else in the private sector. If that happened, the patients typically say they did not understand anything, so they still come to talk with me.

ID6

Ideally, as already mentioned, many of the GPs prefer to see shy and reluctant patients, many of whom have a psychiatric diagnosis, physically with the aim of fostering relational trust and a better working relationship:

[I prefer]...making them leave the house, so that they do not just stay at home and isolate themselves. So, it is better to have psychiatric follow-up consultations at the medical center.

ID18

However, the GPs consider “good enough” remote medical care by a contract GP to represent an acceptable compromise, if the patient specifically prefers it, to maintain some sort of relationship.

### Advantages and Dilemmas of Text-Based Consultations

On the positive side, GPs discussed how text-based consultations have introduced new possibilities. The patient can freely describe his or her reason for contact and include illustrative photographs or videos. In situations with a risk of disease transmission, this can be helpful:

We use that quite a lot; text-based consultations for typical wounds, rashes, and similar things. Most recently I had a chicken pox diagnosis last week. It is nice not to have children with chicken pox in the waiting room.

ID5

Attachment of photographs or videos was also described as effective in the follow-up of a healing process and rehabilitation. GPs can, on their side, use text-based consultations to attach files with ready-made patient information and other instructive material at any stage of the clinical trajectory. They can also ask patients to fill in various forms digitally, including mental health diagnostic checklists or scales, symptom diaries, or text for declarations required by the Norwegian Welfare System, as well as adding self-written parts in the patient record:

That is such a “game changer” for me. It is an incredibly clever new way of doing it, which also makes the patient quite responsible. “Ok, sit down and think about it and write until next time ‘or’ if you are having a headache, write down all about it from the beginning till now.”

ID13

The group dynamics then prompted deliberations on more negative aspects, particularly the dilemma regarding the utilization of predefined questionnaires. These questionnaires encompass a series of clinical inquiries presented in a flowchart format intended to streamline the process, reduce dependence on health secretaries for triage, and aid doctors in promptly addressing the patient’s reason for contact. The general tone was, however, marked by skepticism and call for precaution:

This development [with fixed questions] is happening quickly and quietly without anyone knowing the long-term consequences.

ID18

Participating GPs referred to a well-documented communicative routine in physical, effective patient-centered communication, namely, to invite patients to freely formulate their reason for contact before zooming in on further clinical details:

Letting the patient express him/herself freely is preferable [...] and prefixed algorithmic questions should be used with the greatest caution. It takes away the information we are so used to handling; listening to what the patient says.

ID4

Losing out on the patient’s unfiltered, personal account at the beginning of a consultation might lead to time-consuming diversions and a need to revisit the reason for contact at a later stage. In other words, this created more work for the GP.

In text-based consultations, some patients exaggerate to get attention and skip the line:

Some patients write dramatically as if it were an emergency, yet there is not...

ID15

Another issue of concern related to text-based consultations was that some patients use rough language characterized by profanity and exaggerations, resembling the comment field jargon in social media. GPs worry because the communication is automatically saved in the patient’s medical record, with a potential for unforeseen future implications:

There is a lot of dialect, slang, profanity, and things that are not suitable for the medical record...They can write anything. There are no restrictions.

ID12

Beyond the fear of missing serious disease in a flow of incoming requests, some GPs had noted that informal chat-like text-based consultation sequences back and forth between patient and doctor can be difficult to terminate:

It is difficult with text consultations because it turns out to be a running dialogue that goes back and forth all the time, like a tennis match...which I could have ended in a stricter way, but I find it difficult to do it in a gentle way.

ID11

Considering all these factors, participants suggested that remote consultations might augment the GP’s workload.

## Discussion

### Principal Findings

This focus group study among Norwegian contract GPs shows that the rapid implementation of remote consultations elicited diverse effects on clinical communication and interaction for better and for worse. The appropriateness of remote communication appears contingent on context; recognizing the patient’s characteristics and the clinical relationship takes precedence over the reason for contact or the nature of the health problem. Although entailing a definite potential to enhance efficiency, remote modalities also pose a discernible risk of compromising the retrieval of crucial information and unarticulated problems with clinical relevance. Nonetheless, a somewhat unexpected advantage of remote consultations lies in their potential role as relational “icebreakers,” as some patients find it easier to seek help from their GP and broach sensitive topics from a distance than in face-to-face encounters. Text consultations evoke ambivalence among GPs. Although being of beneficial use in relation to clear reasons for contact, their safety hinges on patients’ ability to articulate their complaints. Strategic preservation of continuity of care emerges as crucial in mitigating the identified challenges.

### Findings in Light of Existing Literature

#### Context-Dependent Suitability

A central finding in our study is that the observed changes in communication during remote consultations are not primarily associated with specific reasons for contact. Instead, they are more influenced by the quality of the doctor-patient relationship and the GP’s evaluation of the patient’s individual ability to express health concerns coherently. The context-dependent qualities of remote consultations resonate with the conclusion of health care researcher Greenhalgh et al [62] that dilemmas about establishing remote consultation services cannot be resolved by standard procedures or algorithms but rather by attending to here-and-now practicalities applied with contextual judgment. Our findings also fit with a recent large in-depth study of patient experiences of remote consultations, stating that it can be influenced by previously unreported patient characteristics and the conditions they consult about [63].

#### Optimizing Communication Styles and Workflow

Our research indicates that remote communication can enhance workflow and information gathering, streamlining a portion of the tasks encountered in a doctor’s daily routine. This aligns with previous research highlighting convenience and time savings offered, especially when physical examination is unnecessary [20-22,63]. Our participating contract GPs reported how, during normal investigations, they could leverage patient-provided information, symptom-scoring forms, and other digitalized documentation more effectively than previously documented [48,52]. However, we also found that remote consultations could potentially increase the overall workload, in accordance with recent research findings [28,33,55]. In total, this paints a nuanced perspective on workflow dynamics and applicability. Ideally, GPs can blend various modalities, leveraging their respective strengths, integrating the straightforward “one-problem approach” with a more comprehensive exploration of the complexity and wider context as needed.

#### Communicative Threats to Quality of Care

Alongside the aforementioned benefits, we identified concerns about unintended loss of contextual information, which is crucial for gaining a comprehensive understanding of more complex health problems. Disregarding life stressors and sociocultural factors might result in degraded clinical decision-making, imprecise or missed diagnoses, and underestimation of severity, findings that expand on prior research [63-69]. This is consistent with research suggesting that remote communication might lead to heightened clinical uncertainty [28-30]. In summary, these challenges suggest that remote communication not only tends to address fewer issues but may also address each problem with a narrower focus, overlooking certain communicative cues and contextual details. Interestingly, this aligns with patients’ perspectives, which highlights the lack of nonverbal communication [26]. Our research also corresponds with a new “masterclass” [23] on communication skills for remote health care, including openings, active listening, and closing nuances, recognizing the differences from face-to-face consultations.

Previous studies have highlighted the constraints of remote consultations in dealing with social determinants of health [12,31,68]. Our results, in particular GPs’ reports on “the one-problem approach,” indicate that unless the GP actively counteracts the inclination to “narrow down,” less urgent but still significant matters on chronic diseases might go unaddressed. This situation calls to mind a noteworthy 1979 paper by GP academic Nigel Stott, who highlighted the distinctive opportunities for including the management of continuing problems and providing some opportunistic preventive health care during routine consultations initiated for other reasons [69]. Our findings suggest an untapped potential for preventive health care on digital platforms. In particular, text consultations give patients more time to formulate nuanced questions, making them well-suited for meeting the needs of patients with complex and chronic conditions over time, in accordance with previous studies [44,45]. Combining informative materials from reputable health libraries with personalized guidance might enhance understanding and patient engagement. However, effective written consultations rely on patients’ ability to articulate their concerns [45].

Simultaneously, GPs in our study describe challenges associated with unfiltered, unfocused, and even verbally profane written patient accounts. Patients may invertedly divert their problems away from the most adequate management, delaying the detection of serious conditions and limiting care planning [25-29]. In addition, our contract GPs reported making concessions to “please the customer” when selecting remote modalities as they endeavor to maintain the clinical relationship with vulnerable patients. Overall, our findings underscore the importance of considering precautionary measures to uphold quality in the evolving landscape of remote consultations.

Notably, we found that contract GPs, after having acquired substantial experience with remote consultations, describe themselves as more observant of the multidimensional aspects of communication in traditional physical consultations. They underscore the importance of safeguarding these qualities in the future.

#### Facilitating Relationships With Reluctant Patients

Up to this point, face-to-face consultations have been considered essential for establishing workable doctor-patient relationships [40,70]. Our study confirms the importance of physical consultations but provides a more nuanced picture. Under certain circumstances, remote consultations can foster new alliances with reluctant patients in need of health care who might otherwise not consult at all. Remote consultations can also function as icebreakers, giving patients an opportunity to open sensitive topics. Examples mentioned included histories of sexual abuse and suicidal thoughts that the patient finds hard to share with the GP in a physical encounter. Furthermore, remote consultations can offer expedited, just-in-time relief to well-known, vulnerable, and unstable patients. In these cases, remote communication seems to consolidate trust over time [71-74]. This also covers text-based consultations, extending the insights from prior literature [32,44]. The findings confirm emerging patient perspectives on text consultations, where attitudes vary widely on their suitability for handling sensitive topics [64].

It has been argued that youth and young adults, groups potentially underusing health services for psychosocial challenges, can benefit from remote consultations, although such consultations have been found to be of shorter duration than physical consultations [68,74]. Our GPs note how consultations with youth often proceed in a somewhat casual manner, with a handheld camera, sometimes on the move, bringing to mind the jargon term “snapping.” Similarly, the discourse in text-based consultations with young individuals may “ping-pong” back and forth, resembling a conversational chat*.* Despite such informality, the GP-patient relationship may derive advantages. We propose that the rise in informality during remote communication, driven by technological advancements and societal shifts, attracts new user groups to these modalities. Health care professionals adopt this approach to connect with these specific patient groups.

#### Power, Trust, and Risk

Norwegian philosopher Grimen [71,72] wrote substantially about trust as a fundamentally important phenomenon in clinical relationships, describing a “nexus” of power, trust, and risk. Despite publishing before the era of remote consultations, he underlined that health care is constantly undergoing change and invited others to reflect upon how changes impact the power-trust-risk nexus. We have already discussed how remote consultations might offer new opportunities in relation to certain reluctant and vulnerable patients. At first glance, remote consultations can be seen as empowering patients in general, offering “consumer” freedom. However, if not deliberately chosen as appropriate, the remote mode may undermine the quality of the GP’s work and ultimately damage trust. Contrary to some prior research [9,64], our findings imply caution in granting patients unrestricted choice regarding consultation type.

### Strength and Limitations

The research team, composed of seasoned contract GPs and digital health researchers, ensured a robust and multifaceted approach to the study. Another key strength was the recruitment and participation of experienced GPs representing diverse demographics, including gender, age groups, and remuneration systems. Their practices spanned urban and rural regions across Norway, contributing to a comprehensive understanding of remote consultations in different settings. This purposive sampling provided a wide range of perspectives. Furthermore, conducting focus groups proved particularly effective for exploring attitudes, experiences, and areas with limited prior knowledge. This methodology facilitated the exploration of tentative and conflicting views, allowing for nuanced reflections, examples, and insights. The use of semistructured interview guides, detailed transcription, and reflexive thematic analysis contributed to the depth and reliability of the findings. We believe that we have sufficient information power to answer our research questions [75].

However, one limitation is that the study did not address potential challenges related to ethnic and cultural diversity among patients and doctors [64]. This omission may be partly due to demographic realities in Norway and the tendency of dedicated regular GPs to avoid repeated digital consultations with patients if they encounter language problems or other contextual barriers, thereby having acquired few significant experiences. We also have sparse data from young GPs; this could have added nuance to our results. The study might also have benefited from the formal inclusion of citizen or patient user perspectives [41].

Potential bias from a few participants’ familiarity with the research team may have influenced responses, yet it facilitated more open dialogue. Also, contrary to expectations that remote interviews would reduce data richness, they appeared to yield engaging interactions. The study was not designed to evaluate each consultation modality (ie, video, text-based, and telephone consultations) in depth. It is important to note that the study was conducted during the aftermath of the pandemic, and it is questionable whether enough time had elapsed to establish “the new normal” [12,13]. During the study period, GPs were still receiving full remuneration for telephone consultations, which might have influenced their choices of remote modality. However, since the telephone has long been a contact medium in general practice, participants appeared particularly interested in discussing novel modalities, such as video and text consultations. Finally, the study focused on microperspectives of doctor-patient communication, leaving broader systemic impacts to be addressed in future research.

### Conclusions

The implementation of remote consultations introduces a spectrum of effects on clinical interaction and communication. These modalities can enhance efficiency but also pose risks, such as compromised retrieval of essential information and unvoiced problems, potentially leading to unintended consequences. Preserving continuity of care is crucial to mitigate these challenges.

Knowledge of the patient and the context plays a crucial role in determining when remote consultations are appropriate. For some patients, remote communication may even contribute to building trustful alliances and enhancing the doctor-patient relationship. Future research could delve into the possibilities of proactive health care initiatives during remote consultations and examine the broader ramifications they have on physicians, medical practices, and societal dynamics.

## Appendix

Multimedia Appendix 1 COREQ checklist.

Multimedia Appendix 2 Information and consent scheme/application for privacy and anonymity (same text used for both purposes; translated from Norwegian).

Multimedia Appendix 3 Interview guide (translated from Norwegian).

## Abbreviations

COREQ: Consolidated Criteria for Reporting Qualitative Research
GP: general practitioner

## Data Availability

The datasets generated and analyzed during this study are not publicly available, but access to the anonymized material can be granted upon reasonable request.
