# Supplementary figures and images for: The Dynamics of Doctor-Patient Communication During Remote Consultations: Qualitative Study Among Norwegian Contract General Practitioners
Source: J Med Internet Res. 2025 Mar 27;27:e57679. doi: 10.2196/57679 (PMC11986389; doi:10.2196/57679)

# Multimedia Appendix 1


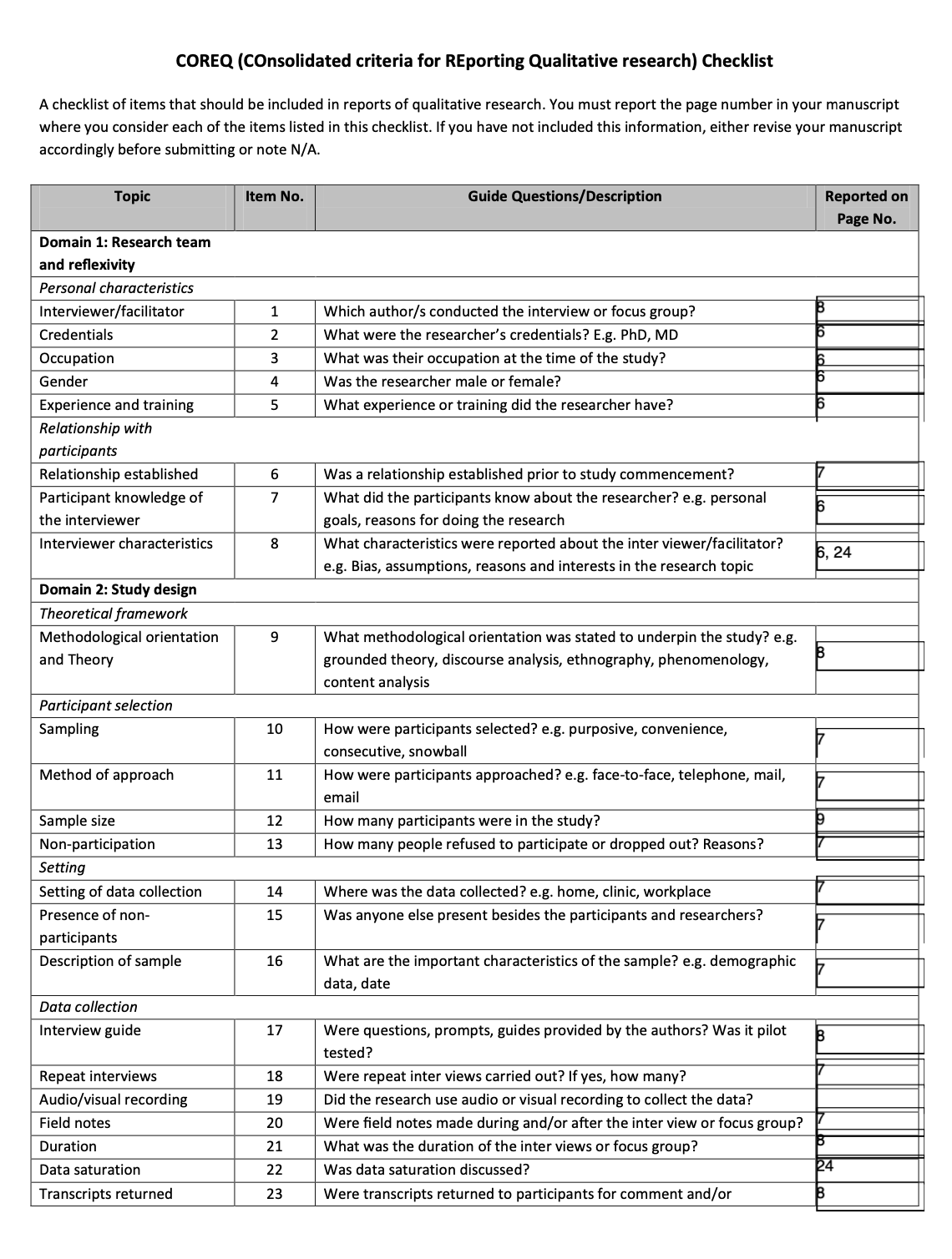


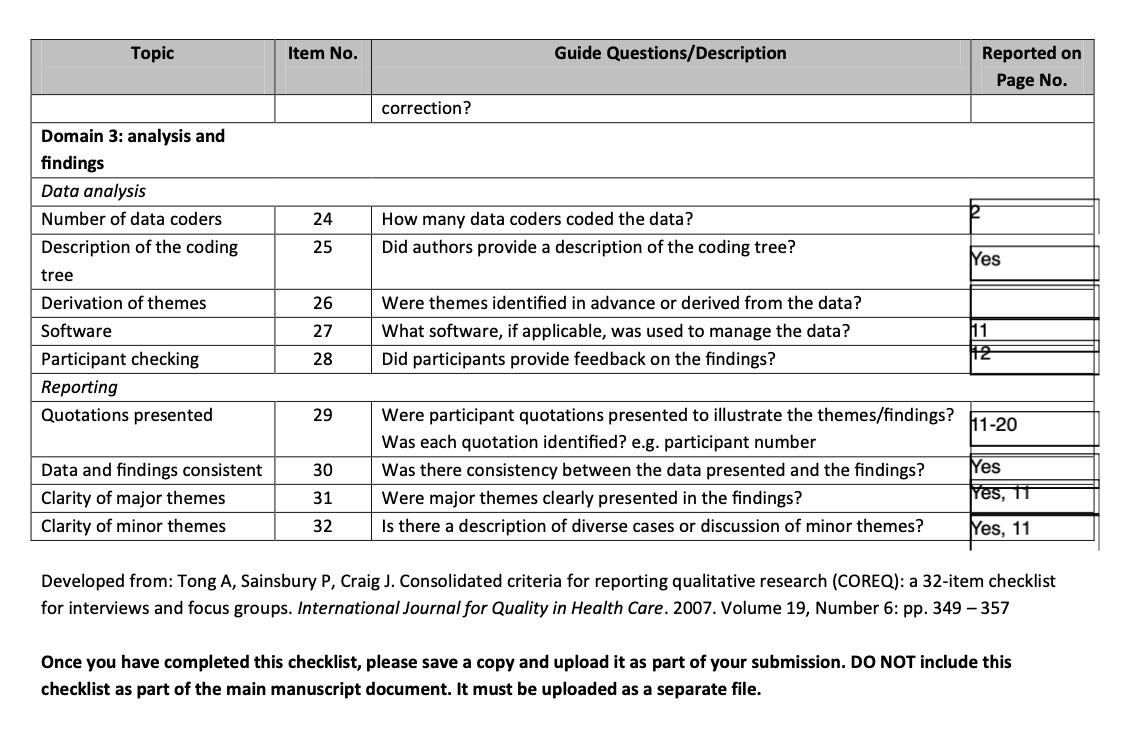

Supplement: Multimedia Appendix 1 [file jmir_v27i1e57679_app1.docx]
