# Supplementary material for: The Dynamics of Doctor-Patient Communication During Remote Consultations: Qualitative Study Among Norwegian Contract General Practitioners
Source: J Med Internet Res. 2025 Mar 27;27:e57679. doi: 10.2196/57679 (PMC11986389; doi:10.2196/57679)
Supplement: Multimedia Appendix 3 [file jmir_v27i1e57679_app3.docx]

# Multimedia Appendix 3

## Interview guide

(Translated from Norwegian)

"Welcome and thank you for participating in this focus group. Our study aims to evaluate remote health services with general practitioners (GPs). Your participation is entirely voluntary, and you may withdraw at any time without any consequences.

We will be recording today's session to accurately capture your insights. Rest assured that your identity will remain anonymous in any reports or publications resulting from this research. All data will be securely stored and kept confidential.

We may ask you, or you may ask us, to review our notes or interpretations of today's discussion to ensure they accurately reflect your views. We have previously conducted other studies in the field, which has helped us ensure the reliability of our research and develop this interview guide.

Please respect each other's perspectives during the discussion and feel free to speak openly. Your insights are invaluable to us. Do not be afraid to express your honest opinions and relate them to your experiences. Different viewpoints enrich the research, making it deeper and more valuable. Thank you again for your participation. Let us begin.

Demographic info, “warm-up questions.”

- What proportion would you estimate digital consultations (text/telephone/video) make up in your practice?
- How does digital *communication affect your everyday working life?*
- Now we have been through a longer pandemic and digitization - Do you see the contours of a new "optimal" way of working?
- Do digital consultations *replace* the physical ones, or do they come in addition?
- Are we losing some of the *"gold"* in general medicine? Is something “human” being lost?
- What is the best/worst things about text consultations?
- What is the best/worst things about video consultations?
- What is the best/worst things about telephone consultations?
- When is it appropriate to utilize digital consultations? For which patients, situations, or types of problems are they most suitable?
- We have heard doctors say that video or text can be a great way to get to know new patients...?
- What communication techniques are employed for video, text, and telephone consultations?
- How is relationship-building facilitated during digital consultations?

*(In Norwegian)*

## Norsk Intervjuguide

Velkommen og takk for at du deltar i denne fokus-gruppen! Denne studien tar sikte på å evaluere digitale helsetjenester (tekst/telefon/video) med fastleger. Din deltakelse er helt frivillig, og du kan trekke deg når som helst uten at det får konsekvenser.

Vi vil gjøre opptak av dagens sesjon for å kunne studere bidragene i etterkant. Alle data vil lagres sikkert og holdes konfidensielt. Du kan også være trygg på at identiteten din vil forbli anonym i alle rapporter eller publikasjoner som er et resultat av denne forskningen. Vi kan be deg, eller du kan be oss om å gjennomgå notatene eller tolkningene våre av dagens diskusjon for å sikre at de gjenspeiler dine synspunkter nøyaktig. Vi har tidligere utført andre studier innen feltet, noe som har hjulpet oss med å sikre påliteligheten til forskningen vår og utvikle denne intervjuguiden.

Vennligst respekter hverandres synspunkter og perspektiver under diskusjonen, og snakk gjerne åpent. Din innsikt er verdifull for oss! Ikke vær redd for å uttrykke dine ærlige meninger og relatere dem til dine erfaringer. *Ulike* synspunkter beriker forskningen, og gjør den dypere og mer verdifull. Takk igjen for din deltakelse! La oss begynne.

- Demografisk informasjon og oppvarmingsspørsmål.
- Hvor stor andel vil du anslå at digitale konsultasjoner (tekst/telefon/video) utgjør i praksisen din?
- Hvordan påvirker digital kommunikasjon arbeidshverdagen din?
- Nå har vi vært gjennom en lengre pandemi og digitalisering – Ser du konturene av en ny «optimal» måte å jobbe på?
- Erstatter digitale konsultasjoner de fysiske, eller kommer de i tillegg?
- Mister vi noe av «gullet» i allmennmedisin? Mister vi noe mellommenneskelig?
- Hva er det beste/verste med tekstkonsultasjoner?
- Hva er det beste/verste med videokonsultasjoner?
- Hva er det beste/verste med telefonkonsultasjoner?
- Når er det hensiktsmessig å bruke digitale konsultasjoner – for hvilke pasienter, situasjoner og type problemer passer det best?
- Vi har hørt leger si at video eller tekst kan være en fin måte å bli kjent med nye pasienter på...?
- Hvilke kommunikasjonsteknikker bruker du i video/tekst/telefonkonsultasjoner?
- Hvordan skjer relasjonsbygging under digitale konsultasjoner?
